# Germline mutations in apoptosis pathway genes in ovarian cancer; the functional role of a TP53I3 (PIG3) variant in ROS production and DNA repair

**DOI:** 10.1038/s41420-021-00442-y

**Published:** 2021-03-29

**Authors:** Sophia R. Chaudhry, Jaime Lopes, Nancy K. Levin, Hasini Kalpage, Michael A. Tainsky

**Affiliations:** 1 Center for Molecular Medicine and Genetics, Wayne State University School of Medicine, Detroit, MI USA; 2 Department of Laboratory Medicine and Pathology, Mayo Clinic, Rochester, MN USA; 3 Department of Oncology, Wayne State University School of Medicine, Detroit, MI USA; 4 Molecular Therapeutics Program, Karmanos Cancer Institute at Wayne State University School of Medicine, Detroit, MI USA

**Keywords:** Cancer genetics, Diseases

## Abstract

Approximately 25% of all cases of ovarian cancer (OVCA) cases are associated with inherited risk. However, accurate risk assessment is limited by the presence of variants of unknown significance (VUS). Previously, we performed whole-exome sequencing on 48 OVCA patients with familial predisposition, yet negative for pathogenic *BRCA1/2* mutations. In our cohort, we uncovered thirteen truncating mutations in genes associated with apoptosis (~35% of our patient cohort). The TP53I3 p.S252X premature stop gain was identified in two unrelated patients. *TP53I3* is transcriptionally activated by p53 and believed to play a role in DNA damage response and reactive oxygen species-induced apoptosis. In addition, nonsense variants in apoptosis-related genes *TP53AIP1*, *BCLAF1*, and *PIK3C2G* were identified in our cohort; highlighting the potential relevance of genes involved in apoptotic processes to hereditary cancer. In the current study, we employed functional assays and demonstrated that cells expressing TP53I3 p.S252X displayed decreased homologous recombination repair efficiency and increased sensitivity to chemotherapeutic drugs bleomycin, mitomycin c, and etoposide. In addition, in the presence of oxidative stress from hydrogen peroxide or etoposide we observed a reduction in the formation of reactive oxygen species, an important precursor to apoptosis with this variant. Our findings suggest that the combination of in silico and wet laboratory approaches can better evaluate VUSs, establish novel germline predisposition genetic loci, and improve individual cancer risk estimates.

## Introduction

Ovarian cancer (OVCA) is the fifth leading cause of cancer-related death among women and carries a 5-year survival rate of <50%. The most common subtype of OVCA is epithelial ovarian cancer (EOC), which is often aggressive and diagnosed at later stages^1^. Approximately 25% of all EOC cases are considered to be hereditary but this figure is most likely an underestimation owing to missing heritability^2,3^. Risk factors for hereditary breast and ovarian cancer syndrome (HBOC) include a family history of ovarian and/or breast cancer, Ashkenazi Jewish heritage, early age of onset, presence of *BRCA1/2* mutations, and mutations in other DNA repair genes or mutated mismatch repair genes also associated with Lynch syndrome^4^. Owing to the high heritability, all OVCA patients are recommended to undergo genetic testing for a panel of at least 25 genes involved in DNA repair, cell cycle regulation, cell adhesion, and RAS signaling. However, panel gene testing is limited because much of the underlying genetic risk is unexplained. In addition, a large fraction of HBOC variants are uncharacterized or of unknown significance (VUS), meaning the effect on protein function, therefore, contribute to disease occurrence is not understood^5^. VUSs and novel genetic loci are the main contributors to the issue of missing heritability in HBOC. This phenomenon limits a clinician’s ability to properly counsel patients on their true genetic risk of HBOC. Therefore, the issue of missing heritability is likely a contributing factor to the mortality rate of OVCA remaining the same for the past 20 years.

A more comprehensive approach to assessing individuals with suspected HBOC risk is whole-exome sequencing (WES)^6^. Clinicians can gain a better understanding of the genetic profile of patients, identify novel risk loci outside of the standard genetic panels, and have the ability to re-visit the data in the future^6^. We have previously reported the discovery of novel genetic risk loci in the WES of germline DNA of OVCA patients^7,8^. Five of the mutations were clinically actionable and an additional 11 high-impact variants that might contribute to cancer development were identified^7,8^. Of particular interest is the rare p.S252X (rs145078765, MAF = 0.0009) premature stop gain mutation in tumor protein p53 inducible protein 3 (TP53I3).

*TP53I3*, formally known as *PIG3*^9^, is unique because it is a quinone oxidoreductase (QOR)^10^, involved in both the DNA damage response^11,12^, and p53-mediated apoptosis^13,14^. Identifying this truncation in *TP53I3* resulted in the extension of our in silico SNP assessment to include genes that are part of the conserved programmed cell death pathway, apoptosis. The *TP53I3* p.S252X variant (rs145078765, MAF = 0.0016) is a nonsense mutation upstream of three residues important in maintaining the binding affinity for QOR substrates, such as naphthoquinone. When a QOR substrate is bound to the enzyme at the active site, reactive oxygen species (ROS) are produced in order for damaged cells to undergo apoptosis. We explored the possibility of *TP53I3* p.S252X, affecting ROS production and potentially downstream apoptotic events.

## Results

### Identifying novel risk loci in apoptosis genes

**Fig. 1:**
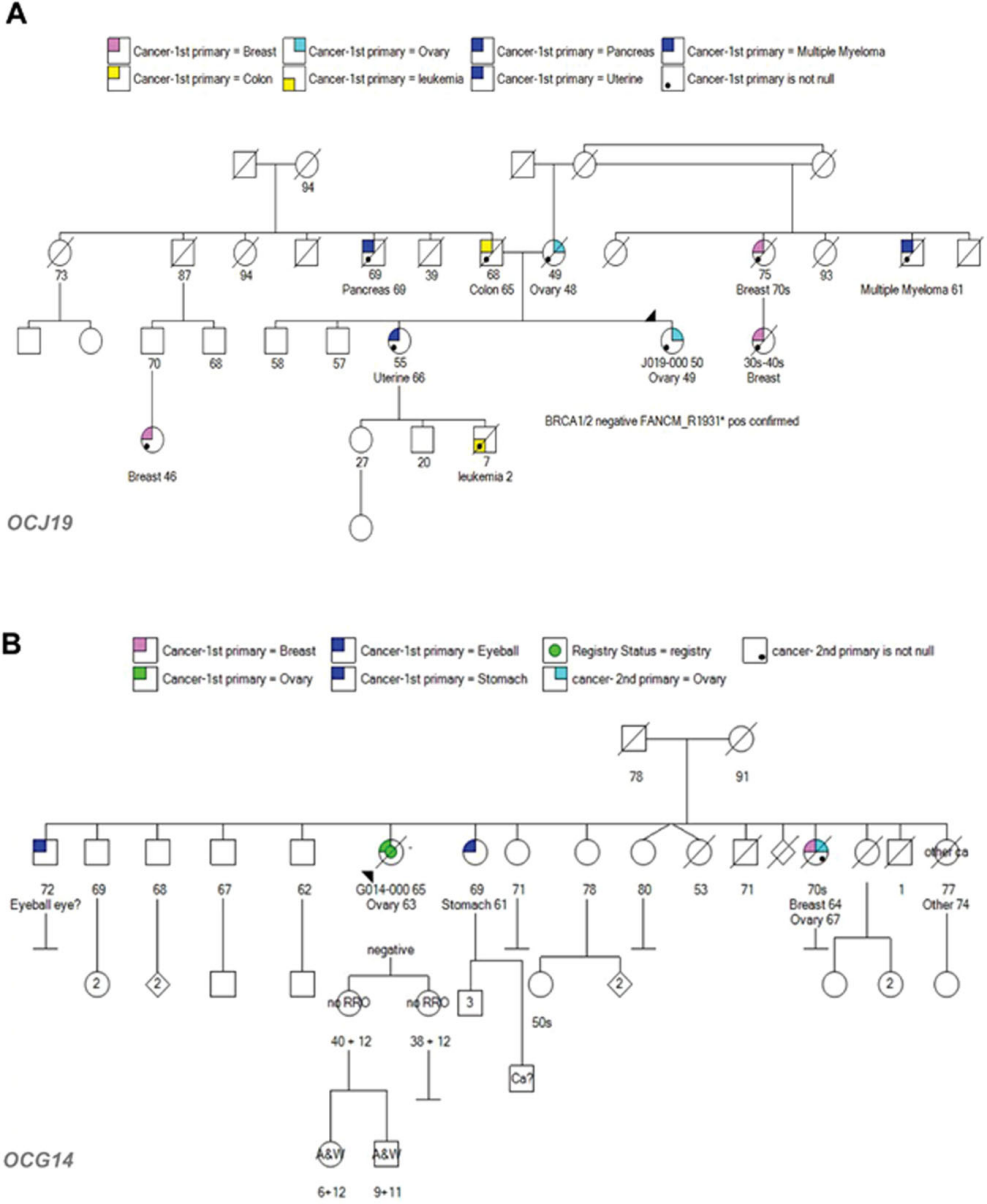
Patient pedigrees for carriers of TP53I3-S252X. **A** The TP53I3-S252X and FANCM-R1931X mutation found in the OCJ19 proband (arrow) and has a family history of breast and ovarian cancer. **B** Patient OCG14 also has a family history of breast and ovarian cancer. No RRO represents no surgical prevention intervention and A&W means the relative is alive and well.

We previously reported the novel risk loci *TP53I3* p.S252X in two of 48 HBOC patients in our cohort, at a higher than expected frequency based on the MAF^7^. The point mutation of a cytosine at position 755 to guanine in the DNA sequence results in a premature stop gain. This nonsense mutation was identified in two unrelated patients OCG14 and OCJ19 (Fig. 1 A and B). In both patients’ family pedigrees, there were multiple incidences of ovarian, breast, prostate, pancreatic, stomach, and myeloma cancers (Fig. 1A and B). The proband OCJ19 also carries the *FANCM* p.Arg1931X (rs144567652) truncation^7^ and two family members who were previously diagnosed with pancreatic cancer or multiple myeloma (Fig. 1A). Several studies indicate that this *FANCM* variant affects the progression of a variety of cancers including breast^15^, non-small cell lung cancer (NSCLC)^16^, colon^17^, and papillary thyroid^18^. TP53I3 overexpression results in a significant increase in breast cancer survival^15^, whereas loss of TP53I3 expression promotes NSCLC, colon, and papillary thyroid cancer^16–18^. To date, there are no germline variants in *TP53I3* associated with cancer risk. We then expanded our query to identify novel germline SNPs in any apoptosis gene and found several of the truncating mutations present in more than one of the 48 HBOC patients (Table 1). Of particular interest were the presence of high-impact mutations in TP53AIP1, BCLAF1, and PIK3C2G in multiple patients.

**Table 1:** Candidate risk mutations in apoptosis genes.

Gene	Consequence	Amino acid	Exon	SNP ID	MAF	OBS
*PIK3C2G*	STOP LOST	*1446Ser	32/32	rs61757718	0.017	2
*TP53I3*	STOP GAIN	Ser252*	4/5	rs145078765	0.0016	2
*TP53AIP1*	FRAMESHIFT	Gln22fs	3/4	rs141395772	0.007	1
*TP53AIP1*	STOP GAIN	Ser32*	3/4	rs140191758	0.0009	1
*BCLAF1*	STOP GAIN	Glu403*	5/13	rs61731960	0.007	1
*BCLAF1*	FRAMESHIFT	His847fs	2/13	rs140096922	0.0003	3
*PPP1R15A*	STOP GAIN	Glu160*	2/3	rs139708522	0.006	1
*DOCK1*	FRAMESHIFT	Asp248fs	8/52	rs768625958	N/A	1
*NLRP1*	FRAMESHIFT	Arg138fs	2/17	rs771551366	0.00007	1
*PTH*	STOP GAIN	Lys85fs	1/3	N/A	N/A	1
*ANGPTL4*	FRAMESHIFT	Gly275fs	6/7	rs747940485	0.0002	1
*NOD2*	STOP GAIN	Trp289*	4/12	N/A	N/A	1
*GZMM*	STOP GAIN	Gln161*	4/5	rs200398398	0.014	1

From left to right; gene name, consequence is the type of mutation, amino acid indicates the translated amino-acid change owing to nonsense (X) or frameshift (fs) mutation, locations of truncations are indicated by Exon, SNP ID designated by dbSNP ID, MAF according to GnomAD non-Finnish Caucasian population minor allele frequency and OBS is the number of patients in the HBOC cohort carrying the truncation among the 48 subjects.

Tumor protein TP53 regulated apoptosis-inducing protein 1 (TP53AIP1) is a mitochondrial protein associated with p53-mediated apoptosis. TP53AIP1 is involved in releasing cytochrome c from the mitochondria and interacts with BCL-2l^19,20^. Diminished expression of TP53AIP1 is associated with increased progression of NSCLC malignancy^21–23^. Two of the HBOC patients carry either the p.Gln22fs truncation (rs141395772, MAF = 0.007) or nonsense mutation p.Ser32X (rs140191758, MAF = 0.0009) in TP53AIP1. These two mutations have been identified in cutaneous melanoma patients^24^. There is conflicting information on whether the two truncations should be considered risk factors for prostate cancer^25,26^.

Three of the HBOC patients carry the same nonsense mutation in BCL-2-associated transcription factor 1 (*BCLAF1*) (rs61731960, p.Glu403X MAF = 0.007) and another patient has a frameshift mutation (rs140096922, H847fs MAF = 0.0003) in that gene. *BCLAF1* is a tumor suppressor that communicates with antiapoptotic members of the BCL-2 family^27^. The BCLAF1 p.Glu403X nonsense mutation that was found in our study was recently identified in four unrelated individuals of a larger population study on germline and somatic variants in OVCA patients^28^. Functional assays found that colon cancer cells expressing wildtype BCLAF1 injected into nude mice caused a decrease in tumor incidence and tumor formation^29^.

Another mutation over-represented in the HBOC cohort was p.X1446Ser stop-loss mutation in phosphatidylinositol-4-phosphate 3-kinase catalytic subunit type 2 gamma (*PIK3C2G*), part of the phosphoinositide 3-kinase (PI3K) family. *PIK3C2G* is an isoform of class II PI3Ks and a tumor suppressor. In the case of colorectal cancer, low copy number variants in *PIK3C2G* resulted in an increased recurrence and poor survival^30,31^. So far, five SNPs in *PIK3C2G* have been significantly associated with HbA1c and/or insulin levels^31^; patients with diabetes have a decreased overall survival rate of OVCA^32^.

### TP53I3-S252X significantly reduces homologous recombination repair (HRR)

**Fig. 2:**
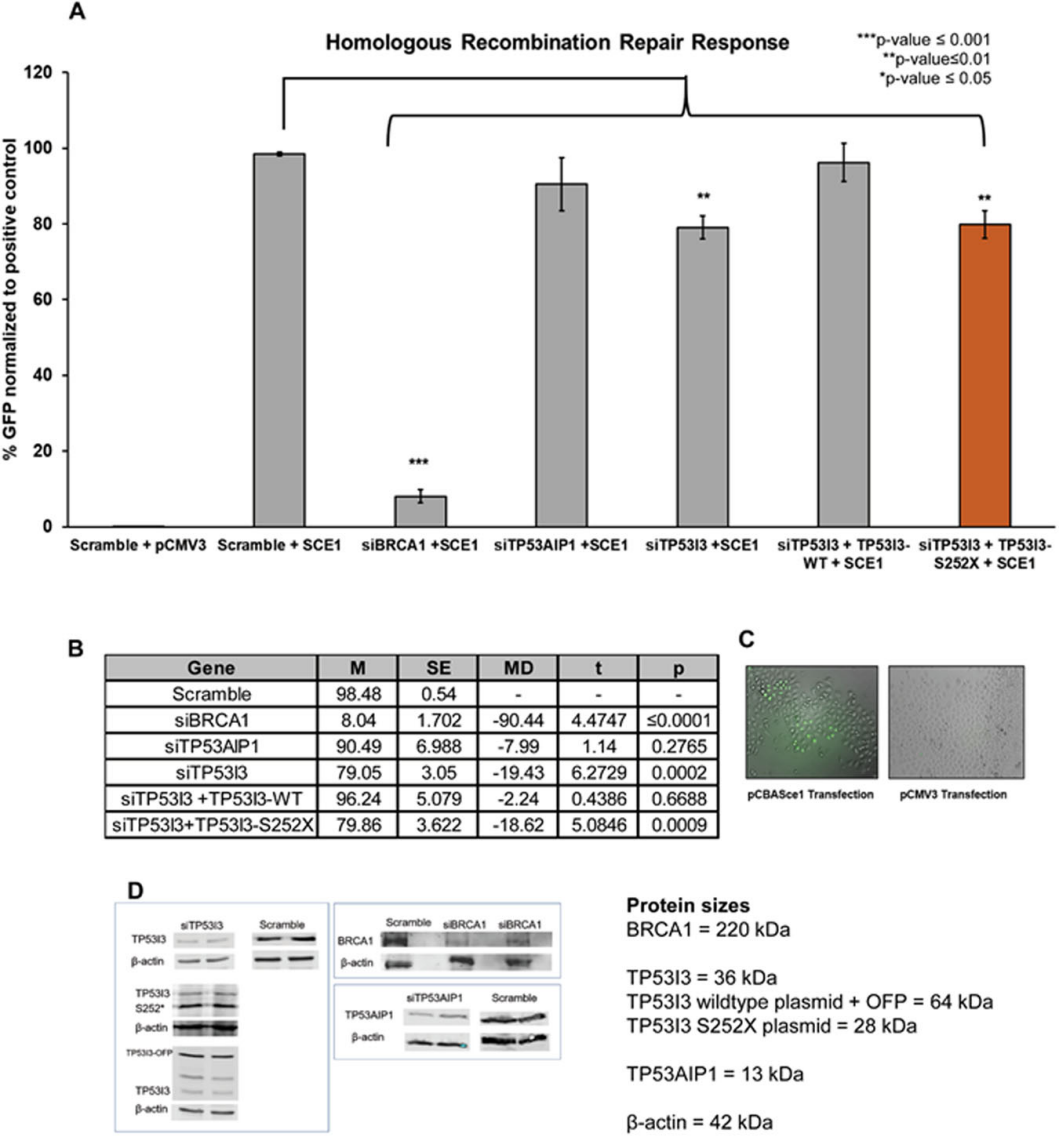
Effect of TP53I3-S252X on homologous recombination repair. HeLa-DR-GFP cells carry two inactive GFP alleles, the first allele contains the SCE1 endonuclease and the second allele is truncated. The introduction of pcBASce-1 to the cells causes a double-stranded break in the first GFP allele and the second GFP allele acts as a template for the HRR of the lesion. GFP expression is a quantifiable measurement of HRR using flow cytometry. **A** Effect of siRNA knockdown of BRCA1, TP53AIP1, TP53I3, or TP53I3 with wildtype (TP53I3-WT) or mutant plasmid (TP53I3-S252X). **B** Calculated two-way *t* test for each condition compared with scramble-positive control. With an *n* = 7, *p* values ≤ 0.05 were considered statistically significant. **C** Fluorescent imaging of HeLa-DR-GFP cells repairing DSB with HRR compared to empty vector pCMV3. **D** Representative western blots for knockdown of TP53I3, TP53I3 rescue with wildtype, TP53I3 rescue with mutant (TP53I3-S252X), BRCA1, and TP53AIP1. The significance test was based on comparing the positive control, Scramble siRNA with SCE1 plasmid, to knockdown and/or rescue conditions.

**Fig. 3:**
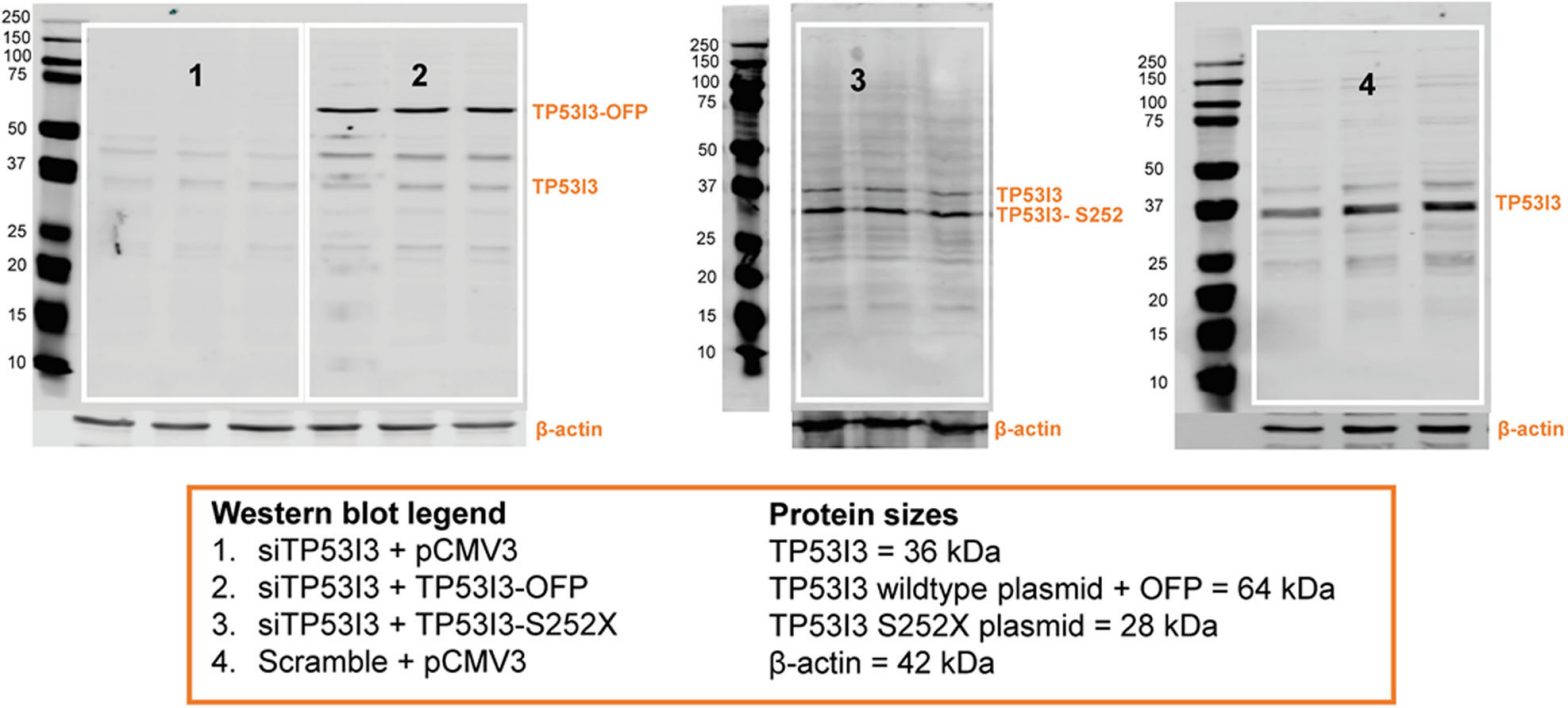
TP53I3 rescue with wildtype and TP53I3-S252X mutant plasmids. For TP53I3 wildtype and mutant expression, HeLa-DR-GFP cells were plated in 60 mm plates with 2 mL of growth media and treated with a transfection complex in parallel with each homologous recombination repair assay. The complex contained 525 pmole TP53I3/scramble siRNA, 5 ng/µL TP53I3 wildtype/TP53I3-S252X/pCMV3 plasmids, 150 µL jetPrime© Buffer, and 9 µL of jetPrime© transfection reagent. TP53I3 was successfully rescued after siRNA knockdown (1) with the TP53I3 wildtype plasmid (2). The wildtype plasmid contains an orange fluorescence protein (OFP) at the C-terminus. TP53I3-S252X mutant expression (3) was successful after transfection into TP53I3-deficient cells. The immunoblot for TP53I3-S252X expression and the markers are from the same gel, with three lanes removed owing to loss of cell lysates volume. At last, endogenous TP53I3 expression was also determined after cells were given Scramble siRNA and the empty plasmid vector pCMV3 (4). Each condition was completed in triplicate to ensure consistency and efficiency of transfections.

HBOC genetic panels typically include *ATM, BRCA1, BRCA2, CHEK2, PALB2, RAD51D*, and *RAD50*, all of which have been associated with the HRR mechanism and risk of HBOC. Therefore, although TP53I3 is normally associated with apoptosis, we tested its effect on DSB DNA repair using HeLa-DR-GFP cells^33^, in which GFP expression is a proxy for HRR occurring in the cells. As we have previously reported, knockdown of TP53I3 with siRNA results in a significant reduction in HRR capabilities^8^. Consistent with our previous findings, knockdown of TP53I3 significantly reduced HRR by an average of 20% (*p* value ≤ 0.01), compared with the positive control (Fig. 2, Supplemental Fig. 1) in our current study. We established the optimal concentration of 5 ng/µL wildtype and p.S252X mutant plasmid was sufficient to rescue TP53I3 after siRNA knockdown and comparable to endogenous protein (Fig. 3). Although HRR was successfully rescued after knockdown and transfection with the exogenous wt-TP53I3 vector, HRR levels in similar experiments with TP53I3-S252X failed to exceed the HRR levels of the CMV vector alone. This indicates that the presence of the truncation negatively impacts HRR activity (Fig. 2, *p* value ≤ 0.01). Conversely, the depletion of TP53AIP1 with siRNA did not significantly reduce HRR. This is consistent with the primary function of the TP53AIP1 protein in p53-mediated apoptosis and maintaining the mitochondrial membrane potential^19^.

### TP53I3-S252X confers significant resistance to mitomycin C and etoposide

**Fig. 4:**
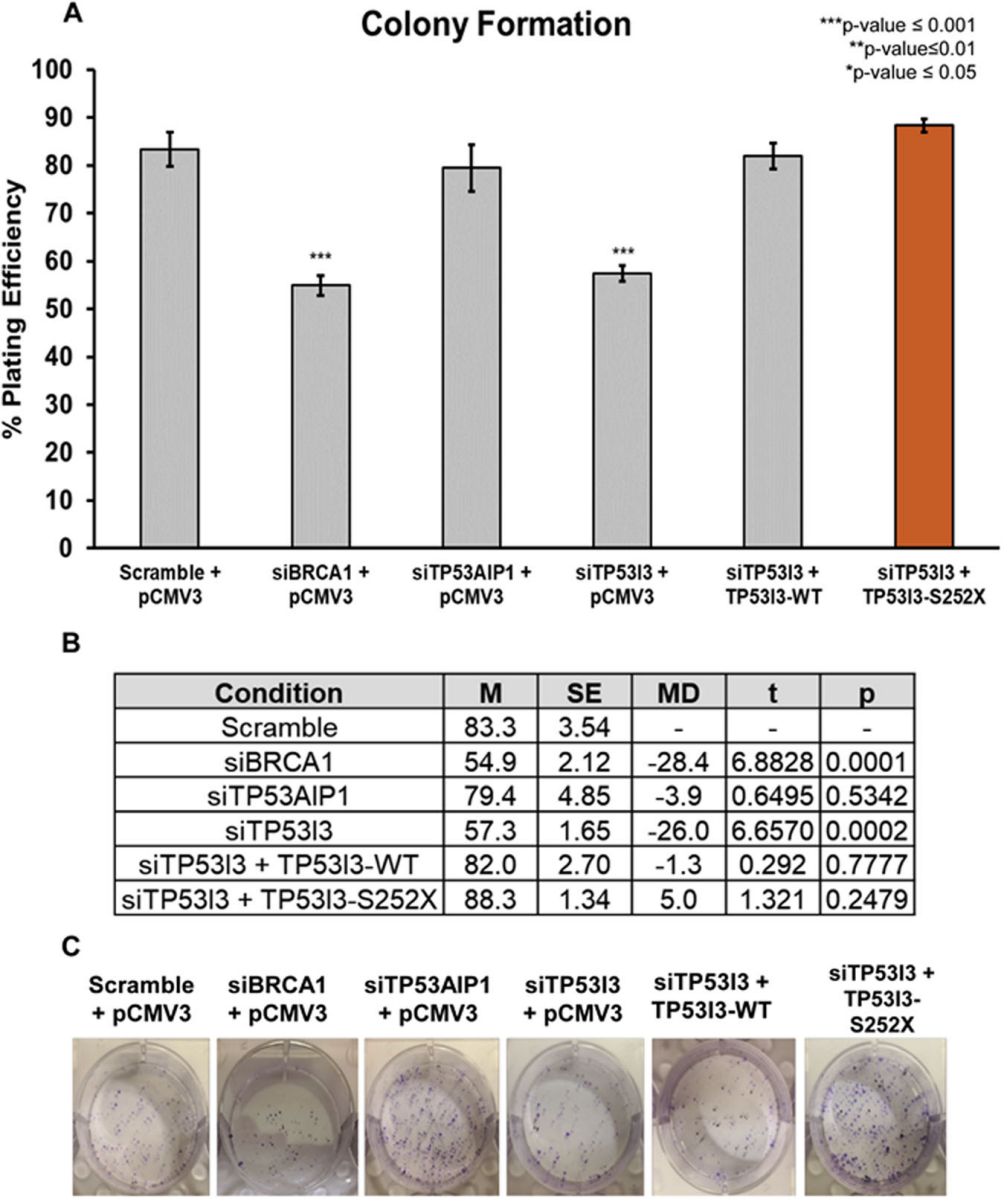
Effect TP53I3-S252X and TP53AIP1 on cell colony formation. **A** The percent plating efficiency on the *y* axis after knockdown of proteins with empty vector (pCMV3). The population of TP53I3 knockdown cells was rescued with TP53I3-WT or TP53I3-S252X mutant. **B** Calculated two-way *t* test for each condition compared with scramble-positive control. With an *n* *=* 5, *p* values ≤ 0.05 were considered statistically significant. **C** Representative qualitative images of each condition. The significance test was based on comparing the positive control, Scramble and SCE1, to knockdown and/or rescue conditions.

**Fig. 5:**
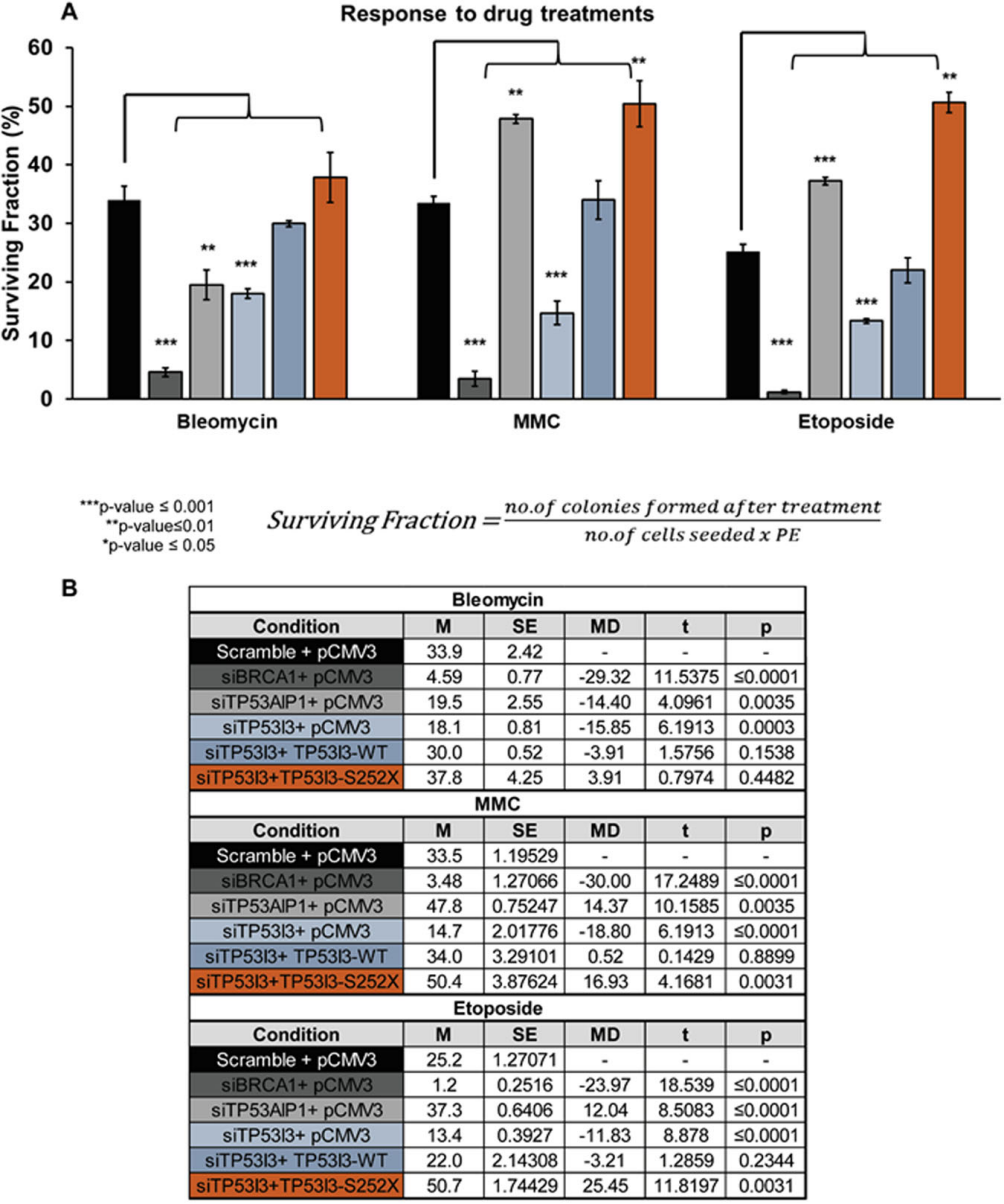
Effect of TP53I3-S252X response to chemotherapy agents. **A** Surviving fraction (*y* axis) after treatment with bleomycin, MMC, or etoposide (*x* axis) incorporates plating efficiency. **B** Calculated two-way *t* test for drug-conditioned cells compared with scramble-positive control. With an *n* *=* 5, *p* values ≤ 0.05 were considered statistically significant.

To determine the sensitivity of cells carrying the TP53I3-S252X compared with the wt-TP53I3 to chemotherapy agents often administered to HBOC patients, we employed a clonogenic assay using HeLa cells. The treatments that we included were mitomycin c (MMC), bleomycin, or etoposide. Depletion of *BRCA1* was used as an internal control to ensure siRNA knockdown affects the cell’s ability to survive after drug treatment. For drug treatments, IC50 dosages of 100 nM for mitomycin C (MMC), 1.5 µM for bleomycin, and 4 µM for etoposide, respectively were employed. In the absence of these cytotoxic agents, knockdown of BRCA1 and TP53I3 exhibited, 28% and 26%, respectively, in the loss of viable clones (*p* value ≤ 0.001, Fig. 4). Approximately 60% of the cells survived after endogenous TP53I3 knockdown using siRNA, compared with the 80% in the scramble control (*p* value ≤ 0.001). Similar to the scramble control, about 80% of cells survived after the rescue of TP53I3 with the wildtype or the mutant TP53I3-S252X plasmid (Fig. 4). Loss of TP53I3 also resulted in the cells being significantly more sensitive to all three drug treatments (Fig. 5, *p* value ≤ 0.05). The rescue of TP53I3 expression after siRNA knockdown with exogenous TP53I3-S252X caused a significant increase in the number of surviving clones after treatment with MMC (*p* value ≤ 0.01) or etoposide (*p* value ≤ 0.01), indicating an acquired resistance of cells bearing this mutation to these DNA damaging agents (Fig. 5).

Knockdown TP53AIP1 cells are resistant to MMC (*p* value ≤ 0.01) and etoposide (*p* value ≤ 0.01, Fig. 5). In contrast, there was a significant decrease in surviving clones after bleomycin treatment (*p* value ≤ 0.01). Bleomycin is a radiomimetic drug that prevents the synthesis of DNA^34^, indicating that a selective sensitivity to this drug by TP53AIP1 could provide the potential for a targeted synthetic lethal therapeutic option.

### TP53I3-S252X increases cell viability in the presence of oxidative stress

**Fig. 6:**
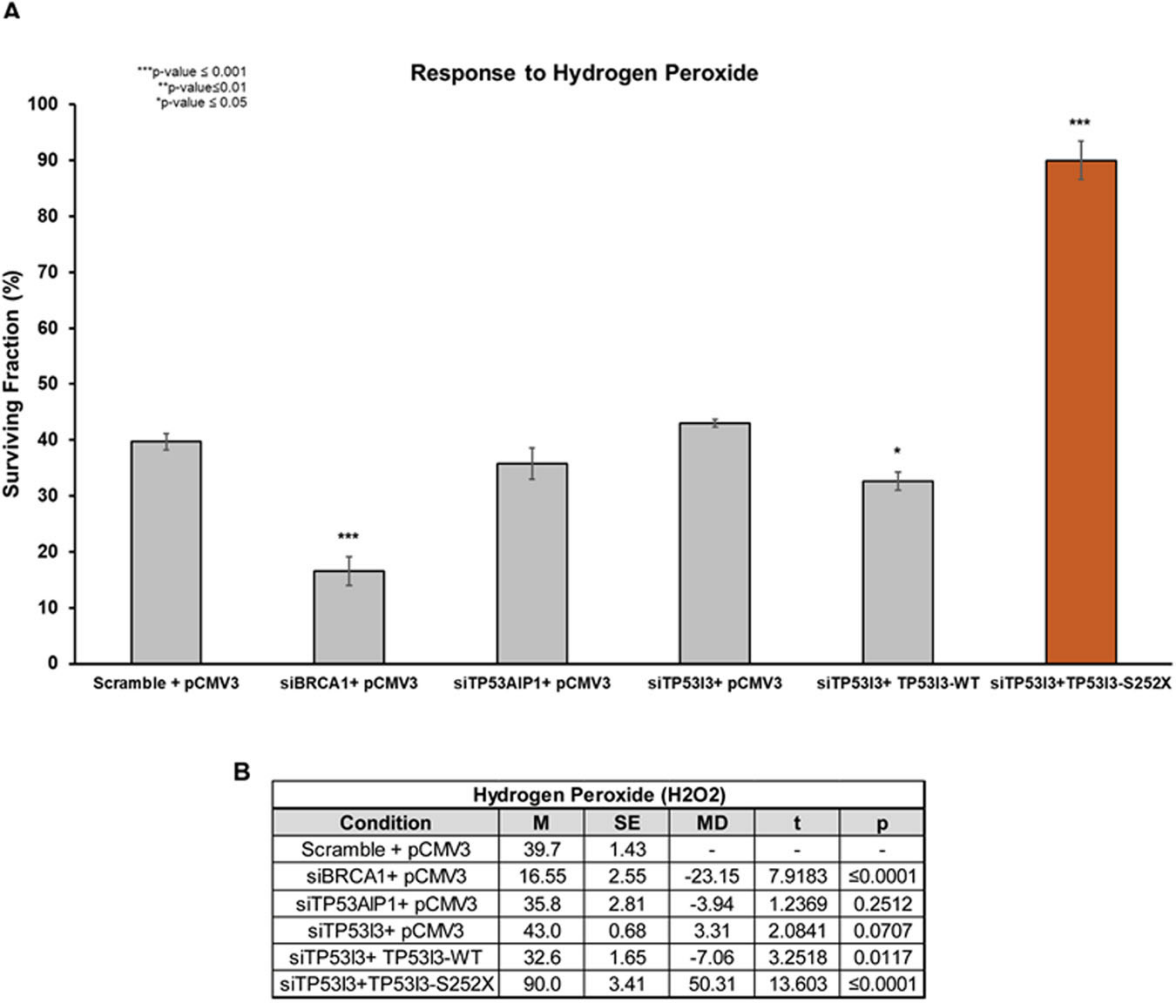
Response of TP53I3-S252X-bearing cells to oxidative stress. **A** Surviving fraction after treating cells with H_2_O_2_ in terms of plating efficiency. **B** Calculated two-way *t* test for H_2_O_2_-conditioned cells compared with scramble-positive control. With an *n* *=* 5, *p* values ≤ 0.05 were considered statistically significant.

Owing to TP53I3’s role in ROS production and p53-mediated apoptosis, the clonogenic assay was also employed to determine whether the p.S252X variant affects the cellular response to oxidative stress. Hydrogen peroxide (H_2_O_2_) can increase levels of superoxide and hydroxyl radicals, leading to ROS production and eventually, apoptosis^35^. Knockdown of TP53I3 in HeLa cells followed by H_2_O_2_ treatment (125 µM), did not change colony formation compared with a scrambled siRNA control (Fig. 6). In contrast, in the presence of TP53I3-S252X after knockdown of the endogenous TP53I3, exposure to H_2_O_2_ resulted in a significant increase in surviving colonies (*p* value ≤ 0.001). This suggested the truncating mutation disrupts a region of the protein that is involved in ROS production. The p.S252X truncation is adjacent to conserved residues that make up the active binding site that interacts with QOR substrates, resulting in the formation of ROS and subsequent apoptosis^10^.

### TP53I3-S252X decreases ROS production

**Fig. 7:**
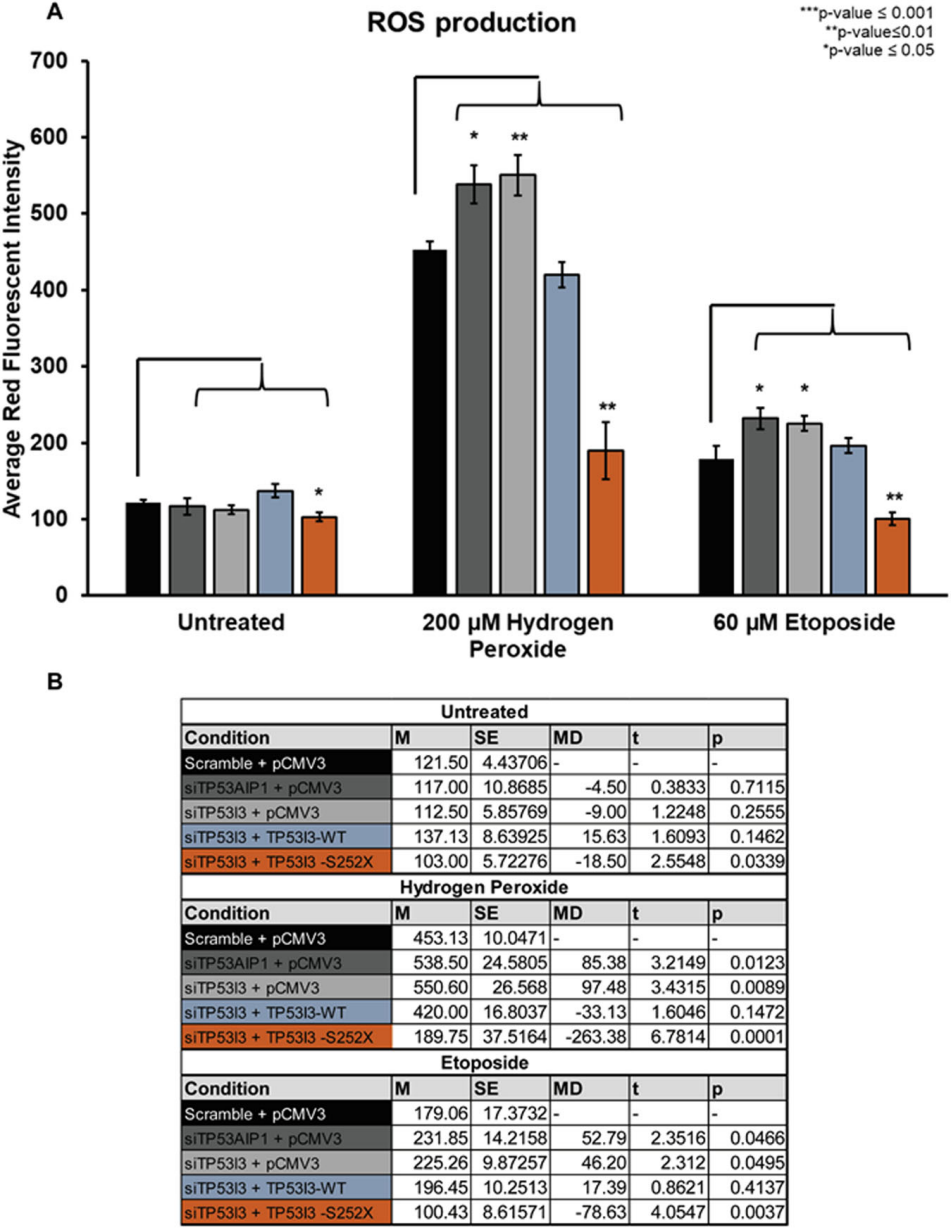
Effect of TP53I3-S252X to mitochondrial ROS production. Red fluorescent intensity (*y* axis) was measured for knockdown of TP53AIP1 or TP53I3 in HeLa cells (without DR-GFP). The population of TP53I3 knockdown cells was rescued with TP53I3-WT or TP53I3-S252X mutant. All conditioned cells were then by treatment with H_2_O_2_ or etoposide. **B** The significance test was based on comparing the positive control, Scramble and SCE1, to knockdown and/or rescue conditions. With an *n* = 5, *p* values ≤0.05 were considered statistically significant.

In order to study this genetic variant further, we analyzed its mechanism of ROS production. The response of *TP53I3-S252X* to MMC, etoposide, and H_2_O_2_ (Figs. 5 and 6) led us to further investigate mechanisms that are used to prevent cell death. *TP53I3* is a member of the QOR gene family that can catalyze the formation of superoxide and hydroxyl ROS. The increase in ROS bodies often signifies the increase in apoptotic cell death. The MitoSox probe is a quantifiable proxy for ROS formation in the mitochondria. Superoxide producing H_2_O_2_ and the topoisomerase II inhibitor etoposide have a well-defined role in ROS production^36^. Both of these agents have the most prominent effect on cell proliferation in the presence of *TP53I3-S252X*. The response to etoposide was of particular interest because of its versatility in treating many types of cancers, including those seen in HBOC high-risk subjects. HeLa cells depleted of TP53I3 (*p* value ≤ 0.01) or TP53AIP1 (*p* value ≤ 0.005) significantly increased the production of ROS after exposure to H_2_O_2_ (Fig. 7). In contrast, after TP53I3 knockdown, we observed a decrease in ROS production in the presence of TP53I3-S252X with treatment with H_2_O_2_ (*p* value ≤ 0.001) or etoposide (*p* value ≤ 0.001). This is likely owing to the fact that the truncation interrupts three downstream residues that are conserved and part of the active binding site that is needed for ROS formation in the presence of ortho-quinone^10^.

## Discussion

### Cell death pathways are important for genetic risk of cancer

In this study, we found that epithelial OVCA patients with a hereditary predisposition and BRCA1/2 wildtype alleles have an enrichment of rare high-impact mutations in apoptosis genes (18 of 48 patients). Many of the truncated genes have already been associated with a variety of cancers. Two of these patients carried SNPs in *TP53AIP1* (rs141395772 or rs140191758) that have been previously associated with melanoma risk^24^; however, these alleles are considered VUSs owing to conflicting reports on the effect of the mutations in prostate cancer^25^. We also observed an overrepresentation of truncations in the *BCLAF1* gene in our cohort, with four patients carrying either rs140096922 or rs61731960. This gene regulates mitochondrial membrane potential and apoptosis^29,37^, and therefore defects in this protein’s function negatively affect a variety of programmed cell death and DNA repair mechanisms. Apoptosis is a tightly controlled and conserved mechanism. Given the large number of truncated apoptosis genes observed in our study, programmed cell death pathways should be considered when examining hereditary cancer families for germline mutations in cancer risk genes.

### TP53I3-S252X disrupts HRR and ROS production

The TP53I3-S252X mutation impeded the proper functioning of DDR, decreased ROS production, and results in resistance to loss of cell reproductive capacity in the presence of cytotoxic agents. This may be related to the fact that cells expressing truncated TP53I3-developing resistance to chemotherapy by using alternative methods of DNA break repair and multiplying, the onset of gene amplification, or disruption of drug transport across the cell membrane, resulting in the inactivation of the drug in the presence of the mutant. The reduction of ROS bodies is similar to the PIG3AS splice variant for *TP53I3* in which exon 4 is spliced out, resulting in an inactive protein^38^. A functional consequence of this natural splice variant is a preferential translation and the disruption of ROS formation. This is owing to the absence of the C-terminal sequence, which is homologous to a QOR, a subclass of the Medium-chain Dehydrogenases/Reductases superfamily^38^. Missense mutations of serine at position 151 to valine in the TP53I3 protein disrupts the conserved binding motif for NADP^+^ (A/G)XXSXXG^39^. This protein is enzymatically inactive due to steric hindrance and therefore a loss of ROS production^10^. Thus, a likely explanation as to why TP53I3-S252X exhibits lower ROS production and cell loss is that there is less or no enzymatic activity.

Three conserved residues distal to the TP53I3 p.S252X truncation, L255, F256, and L265, are part of the protein’s active site^10^. The nonsense mutation prevents the translation of the mRNA sequence after position 252. This means that three of the 15 residues necessary for the enzymatic activity are not present in the TP53I3 protein, thus affecting active site conformation and preventing quinone substrates like 1,2-Naphthoquinone NQ from binding efficiently. In addition, this alteration results in a severe inactivation of the ability to reduce free molecular oxygen to produce ROS species in the presence of a cofactor. This is supported by the fact that TP53I3-S252X cells had a significant decrease in mitochondrial ROS production in the form of H_2_O_2_, which is reduced to hydroxyl radicals^35^ (Fig. 6). Furthermore, in the presence of H_2_O_2_, the TP53I3-S252X transfected cells had a lower colony-forming capacity than wildtype TP53I3-transfected cells (Fig. 6). In the presence of MMC and etoposide, there was a significant increase in the number of surviving cells after transfection of the TP53I3 p.S252X nonsense allele. After the treatment of TP53I3 knockdown cells with the chemotherapy drugs MMC or etoposide, the TP53I3-S252X mutant-transfected cells had lower colony-forming capacity compared with the scramble control (Fig. 5). To maintain normal cellular homeostasis, high levels of cellular ROS should lead to the activation of programmed cell death such as apoptosis. The inability to regulate apoptosis can result in the accumulation of old and damaged cells, which could be a risk-factor for tumorigenesis.

## Methods

### Sample acquisition and tumor histology

We previously studied 48 Caucasian women diagnosed with high-grade serous EOC who did not carry known *BRCA1/2* pathogenic mutations^7^. These patients were selected owing to their personal history of OVCA and suspected hereditary factors of cancer predisposition. For more information about sample acquisition and histology please refer to our previous study^7^.

### WES and candidate gene analysis

Whole-exome sequencing methods, raw data processing, and application of filters to VCF files were performed as in our previous study^7^. Databases and predictive algorithms that were applied included SIFT^40^, PolyPhen^41^, ClinVar^42^, HGMD^43^, COSMIC^44^, and GnomAD^45^.

### Validating SNPs of interest

Variants of interest were confirmed using forward and reverse strand Sanger sequencing (Supplemental Table 1). Mutants were confirmed using QIAGEN Fast Cycling PCR Kit (203743). Amplified genomic DNA from the PCR products was purified using the QIAGEN QIAquick PCR Purification Kit (28106). The reactions were assembled and sent to Genewiz, South Plainfield, NJ, for sequencing and confirmation of the presence of the SNP of interest in the patient.

### Cell culture

HeLa-DR-GFP cells were provided by Dr. Jeffery Parvin from Ohio State University^46,47^. These cells provide the ability to functionally assess the HRR after inducing double-stranded breaks with restriction enzyme pCBASE1 as described^8^.

### Transfection

For protein knockdown, high quality and pure siRNAs were used targeting the 3′-UTR not contained in the plasmid expression vectors (Table 2). Exogenous TP53I3-WT, TP53I3-S252X mutant, and empty vector DNA plasmids were delivered to the cells by transient transfection using jetPRIME Transfection Reagent (Polyplus, Strasbourg, France, 114–15). The concentration of siRNA used for all proteins was 110 pmol/well in a 24-well plate and scaled up to the appropriate cell culture plate when needed.

**Table 2:** Materials for knockdown, protein immunoblotting, and plasmid constructs.

Product	Manufacturer	Catalog no.	Concentration/dilution
Hs_ TP53I3_2 FlexiTube siRNA 20 nmol	Qiagen	SI00069636	110 pmol in a 24-well plate
Hs_BRCA1_13 FlexiTube siRNA 20 nmol	Qiagen	SI02654575	110 pmol in 24-well plate
P53AIP1 siRNA (h) 10 µM	Santa Cruz	SC-37459	110 pmol in 24-well plate
Negative control siRNA 20 nmol	Qiagen	1027310	110 pmol in 24-well plate
PIG3 antibody- mouse (A-5)	Santa Cruz	SC-166664	1:1000 overnight incubation
BRCA1 antibody- mouse (D-9)	Santa Cruz	SC-6954	1:200 overnight incubation
P53AIP1- rabbit antibody	Invitrogen	PA5-20355	1:200 overnight incubation
Beta-actin- rabbit AC-74 antibody	Sigma-Aldrich	A53160-100UL	1:10,000 overnight incubation
Donkey anti-rabbit IgG (H + L) Alexa Fluor® 790	Thermo Fisher	A11374	1:10,000; 1 h incubation
Donkey anti- rabbit IgG (H + L) Alexa Fluor® 680	Thermo Fisher	A10043	1:10,000; 1 h incubation
Donkey anti-mouse IgG (H + L) Alexa Fluor® 790	Thermo Fisher	A11371	1:10,000; 1 h incubation
Donkey anti-mouse IgG (H + L) Alexa Fluor® 680	Abcam	ab175774	1:10,000; 1 h incubation
PIG3 cDNA Clone, human, C-OFPSpark® tag	Sino Biological	HG15531-ACR	N/A

From left to right, the product name according to the manufacturer with catalog number and the amount used for all experiments.

### Cell lysate preparation and western blots

Proteins in cell lysates were solubilized using radioimmunoprecipitation assay buffer (RIPA) with Halt protease (ThermoFisher, 87786) and phosphatase inhibitor cocktails (ThermoFisher, 78420). Quantitation of protein concentration was determined using the DC™ Protein Assay kit (Biorad; 5000113, 5000114, 5000115) and the BioTek Synergy H1 Hybrid Multi-Mode Reader. Cell lysates were prepared with a 4× Li-CORE protein sample buffer (928-40004). The nitrocellulose membranes were incubated overnight at 4 °C with the appropriate primary antibody (Table 2).

### Site-directed mutagenesis

The pCMV3-C-OFPSpark-TP53I3 wildtype expression vector was acquired from Sino Biological (HG15531-ACR). The plasmid contains an open reading frame for the full coding sequence of *TP53I3* followed by an orange fluorescent protein (OFP) marker at the C-terminus. Gene editing of the plasmid was conducted using the Q5-Site-Directed Mutagenesis Kit from New England BioLabs Inc. (E0554S) to create the *TP53I3* 755 C > G, S252X, truncation. Primer sequences were forward 5′-CCCCTGTTTTAAAAAGCTACTTTTTAAG-3′ and reverse 5′-CCCATTGATGTCACCTCC-3′. Endogenous TP53I3 is 36 kDa in size, exogenous TP53I3 is 64 kDa owing to the OFP tag, and the exogenous TP53I3-S252X is 28 kDa. The TP53I3 siRNA targeted a region at the C-terminus, downstream of the truncation 5′-CAGAGCCGTTTAAAGCTGAT-3′.

### HRR assay

To determine the effects of protein knockdowns and mutated proteins on HRR, we employed HeLa-DR-GFP cells^47^. To ensure that the plasmid was not being selected out when passaging cells, 1.5 µg/mL of pusromycin was included in the culture medium. GFP expression was quantified using the BD FACSCanto II at the Wayne State University Microscopy, Imaging & Cytometry Resources (MICR) core. The positive control consisted of pCBASce-1 and scramble siRNA. As a negative control, empty vector pCMV3 was used. A total of 35,000 HeLa-DR-GFP cells were seeded 24 h prior to transfection. The transfection complex consists of pCMV3 empty vector or TP53I3-S252X, wildtype TP53I3, BRCA1 siRNA, or TP53AIP1 siRNA with pCBASceI, and jetprime®PRIME reagent diluted into jetPRIME® Buffer. All conditions were conducted in triplicate for each experiment and raw values were normalized to the positive control. After 72 h, the cells were harvested to quantify for GFP expression.

### Colony survival assay

The clonogenic assay was used to determine a cell’s reproductive capacity after being conditioned with various cytotoxic agents as per our previous work^8^. Conditions for the cells included siRNA knockdown of proteins BRCA1, TP53AIP1, and TP53I3, and, siRNA knockdown of TP53I3 followed by rescue with TP53I3-WT or TP53I3-S252X plasmids. After transfection, 300 cells per condition were plated in triplicate. Drug IC50 concentrations were bleomycin (1.5 µM), mitomycin C (100 nM), etoposide (4 µM), or hydrogen peroxide (H_2_O_2_, 125 µM).

### Mitochondrial ROS production assay

To detect ROS production in a population of HeLa cells under wildtype, we used the Mitosox probe. Mitosox is a positive charge probe to detect superoxide ROS in the mitochondria, which emit red fluorescence (excitation: 510 nm, emission: 580 nm). HeLa cells without the pDR-GFP plasmid were used to avoid interference with the red fluorescence. The transfection complex includes TP53I3-S252X, TP53I3-WT or pCMV3 DNA with siRNA and jetPRIME® reagent. HeLa cells were treated with 60 µM of etoposide or 125 µM of H_2_O_2_ for 4 hours and then stained with 5 µM Mitosox (Invitrogen) for 30 min at 37°C at 5% CO_2_ in the dark. Fluorescence intensity was quantified using the BioTek Synergy H1 Hybrid Multi-Mode Reader.

### Statistical analysis

The values reported in graphs are the mean±standard error from experiments conducted with an *n* of no. <7 and no. >3. A standard two-way student *t* test using GraphPad Prism was conducted to compare all conditions to the positive control. A value of *p* < 0.05 was considered statistically significant.

## Supplementary information

Supplemental Figure 1

Supplemental Table 1

## References

[1] Torre LA (2018). Ovarian cancer statistics, 2018. CA Cancer J. Clin..

[2] Bodmer W, Tomlinson I (2010). Rare genetic variants and the risk of cancer. Curr. Opin. Genet. Dev..

[3] Manolio TA (2009). Finding the missing heritability of complex diseases. Nature.

[4] Saslow D (2012). American Cancer Society, American Society for Colposcopy and Cervical Pathology, and American Society for Clinical Pathology Screening Guidelines for the prevention and early detection of cervical cancer. Am. J. Clin. Pathol..

[5] Richards S (2015). Standards and guidelines for the interpretation of sequence variants: a joint consensus recommendation of the american college of medical genetics and genomics and the association for molecular pathology. Genet. Med..

[6] Chaudhry, S., Stafford, J. L., Tainsky, M. A. & Levin, N. K. Whole exome sequencing: a necessary tool for the future of clinical cancer care. *J. Cancer Biol. Res.* **5**, 1106 (2017).

[7] Stafford JL (2017). Reanalysis of BRCA1/2 negative high risk ovarian cancer patients reveals novel germline risk loci and insights into missing heritability. PLoS ONE.

[8] Lopes JL, Chaudhry S, Lopes GS, Levin NK, Tainsky MA (2019). FANCM, RAD1, CHEK1 and TP53I3 act as BRCA-like tumor suppressors and are mutated in hereditary ovarian cancer. Cancer Genet..

[9] Flatt PM (2000). p53-dependent expression of PIG3 during proliferation, genotoxic stress, and reversible growth arrest. Cancer Lett..

[10] Porté, S. et al. Three-dimensional structure and enzymatic function of proapoptotic human p53-inducible quinone oxidoreductase PIG3. *J. Biol. Chem.* **284**, 17194–17205 (2009). 10.1074/jbc.M109.001800 PMC2719357 19349281

[11] Li B (2013). PIG3 functions in DNA damage response through regulating DNA-PKcs homeostasis. Int. J. Biol. Sci..

[12] Contente, A., Dittmer, A., Koch, M. C., Roth, J. & Dobbelstein, M. A polymorphic microsatellite that mediates induction of PIG3 by p53. *Nat. Genet*. **30**, 315–320 (2002). 10.1038/ng836 11919562

[13] Polyak K, Xia Y, Zweier JL, Kinzler KW, Vogelstein B (1997). A model for p53-induced apoptosis. Nature.

[14] Lee J-H (2010). The p53-inducible gene 3 (PIG3) contributes to early cellular response to DNA damage. Oncogene.

[15] Zhang, W. et al. BRCA1 regulates PIG3-mediated apoptosis in a p53-dependent manner. *Oncotarget* **6**, 7608–7618 (2015). 10.18632/oncotarget.3263 PMC4480703 25797244

[16] Li M (2017). PIG3 promotes NSCLC cell mitotic progression and is associated with poor prognosis of NSCLC patients. J. Exp. Clin. Cancer Res..

[17] Park S-J (2017). The oncogenic effects of p53-inducible gene 3 (PIG3) in colon cancer cells. *Korean*. J. Physiol. Pharmacol..

[18] Xu J (2015). PIG3 plays an oncogenic role in papillary thyroid cancer by activating the PI3K/AKT/PTEN pathway. Oncol. Rep..

[19] Oda K (2000). p53AIP1, a potential mediator of p53-dependent apoptosis, and its regulation by Ser-46-phosphorylated p53. Cell.

[20] Matsuda K (2002). p53AIP1 regulates the mitochondrial apoptotic pathway. Cancer Res..

[21] Fang H (2019). Extracellular vesicle‑delivered miR‑505‑5p, as a diagnostic biomarker of early lung adenocarcinoma, inhibits cell apoptosis by targeting TP53AIP1. Int. J. Oncol..

[22] Yamashita SI (2009). Combination of p53AIP1 and survivin expression is a powerful prognostic marker in non-small cell lung cancer. J. Exp. Clin. Cancer Res..

[23] Yamashita SI (2008). p53AIP1 expression can be a prognostic marker in non-small cell lung cancer. Clin. Oncol..

[24] Benfodda M (2018). Truncating mutations of *TP53AIP1* gene predispose to cutaneous melanoma. Genes, Chromosom. Cancer.

[25] Luedeke M (2012). Prostate cancer risk is not Altered by TP53AIP1 germline mutations in a German case-control series. PLoS ONE.

[26] Wang X (2006). Truncating variants in p53AIP1 disrupting DNA damage-induced apoptosis are associated with prostate cancer risk. Cancer Res..

[27] Cuconati A, White E (2002). Viral homologs of BCL-2: role of apoptosis in the regulation of virus infection. Genes Dev..

[28] Kanchi KL (2014). Integrated analysis of germline and somatic variants in ovarian cancer. Nat. Commun..

[29] Zhou X (2014). BCLAF1 and its splicing regulator SRSF10 regulate the tumorigenic potential of colon cancer cells. Nat. Commun..

[30] Li A (2015). PIK3C2G copy number is associated with clinical outcomes of colorectal cancer patients treated with oxaliplatin. Int. J. Clin. Exp. Med..

[31] Daimon M (2008). Association of the PIK3C2G gene polymorphisms with type 2 DM in a Japanese population. Biochem. Biophys. Res. Commun..

[32] Shah MM (2014). Diabetes mellitus and ovarian cancer: more complex than just increasing risk. Gynecol. Oncol..

[33] Pierce AJ, Johnson RD, Thompson LH, Jasin M (1999). XRCC3 promotes homology-directed repair of DNA damage in mammalian cells. Genes Dev..

[34] Dorr RT (1992). Bleomycin pharmacology: mechanism of action and resistance, and clinical pharmacokinetics. Semin. Oncol..

[35] Bolton JL, Dunlap T (2017). Formation and biological targets of quinones: cytotoxic versus cytoprotective effects. Chem. Res. Toxicol..

[36] Wu, D. & Yotnda, P. Production and detection of reactive oxygen species (ROS) in cancers. *J. Vis. Exp*. 10.3791/3357.(2011). 10.3791/3357 PMC3308605 22127014

[37] Lee YY, Yu YB, Gunawardena HP, Xie L, Chen X (2012). BCLAF1 is a radiation-induced H2AX-interacting partner involved in γH2AX-mediated regulation of apoptosis and DNA repair. Cell Death Dis..

[38] Nicholls CD, Shields MA, Lee PWK, Robbins SM, Beattie TL (2004). UV-dependent alternative splicing uncouples p53 activity and PIG3 gene function through rapid proteolytic degradation. J. Biol. Chem..

[39] Edwards KJ (1996). Structural and sequence comparisons of quinone oxidoreductase, ζ-crystallin, and glucose and alcohol dehydrogenases. Arch. Biochem. Biophys..

[40] Ng PC, Henikoff S (2003). SIFT: Predicting amino acid changes that affect protein function. Nucleic Acids Res..

[41] Adzhubei, I., Jordan, D. M. & Sunyaev, S. R. Predicting functional effect of human missense mutations using PolyPhen-2. *Curr. Protoc. Hum. Genet*. **Chapter 7**, Unit7.20 (2013). 10.1002/0471142905.hg0720s76 PMC4480630 23315928

[42] Landrum MJ (2018). ClinVar: improving access to variant interpretations and supporting evidence. Nucleic Acids Res..

[43] Stenson PD (2017). The Human Gene Mutation Database: towards a comprehensive repository of inherited mutation data for medical research, genetic diagnosis and next-generation sequencing studies. Hum. Genet..

[44] Forbes, S. A. et al. The catalogue of somatic mutations in cancer (COSMIC). *Curr. Protoc. Hum. Genet*. **CHAPTER 10**, Unit 10.11 (2008). 10.1002/0471142905.hg1011s57 PMC2705836 18428421

[45] Karczewski, K. J. et al. Variation across 141,456 human exomes and genomes reveals the spectrum of loss-of-function intolerance across human protein-coding genes. *bioRxiv* https://www.biorxiv.org/content/10.1101/531210v2 (2019).

[46] Parvin, J., Chiba, N. & Ransburgh, D. Identifying the effects of BRCA1 mutations on homologous recombination using cells that express endogenous wild-type BRCA1. *J. Vis. Exp.* **3791** (2019). 10.3791/2468 PMC3197403 21372787

[47] Jasin, M. & Rothstein, R. Repair of strand breaks by homologous recombination. *Cold Spring Harb. Perspect. Biol*. **5**, a012740 (2013). 10.1101/cshperspect.a012740 PMC3809576 24097900

