# Supplementary figures and images for: Germline mutations in apoptosis pathway genes in ovarian cancer; the functional role of a TP53I3 (PIG3) variant in ROS production and DNA repair
Source: Cell Death Discov. 2021 Mar 29;7:62. doi: 10.1038/s41420-021-00442-y (PMC8007802; doi:10.1038/s41420-021-00442-y)

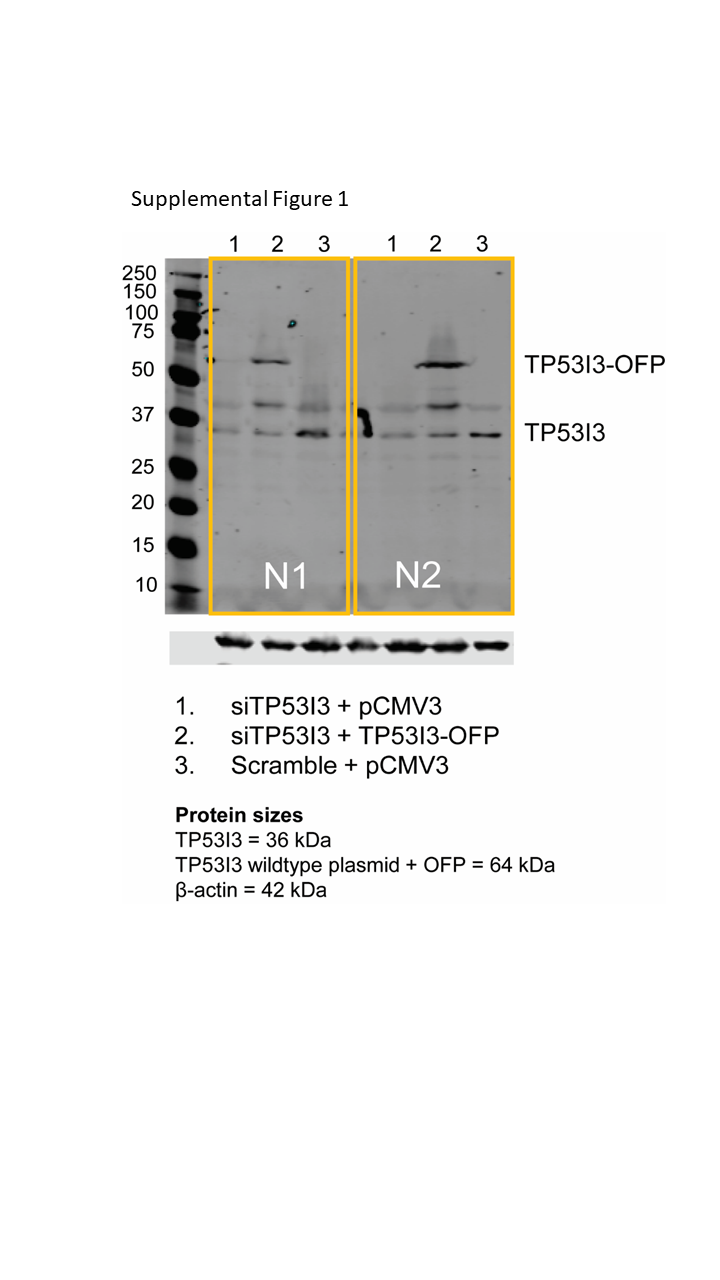

Supplement: Supplementary file 1 — Supplemental Figure 1 [file 41420_2021_442_MOESM1_ESM.png]
